# Diagnostic Approach to Abnormal Urine Colors: Lessons From a Case of Blue-Green Urine

**DOI:** 10.7759/cureus.82122

**Published:** 2025-04-11

**Authors:** Carson Balen, Zayd Chishti, Jason W Wilson

**Affiliations:** 1 Emergency Medicine, University of South Florida Morsani College of Medicine, Tampa, USA; 2 Medicine, University of South Florida Morsani College of Medicine, Tampa, USA

**Keywords:** abnormal urine, abnormal urine color, blue-green urine, methylene blue treatment, urine color change

## Abstract

Abnormal urine coloration can be a perplexing finding in clinical practice, often prompting concern among both patients and healthcare providers. While cases of red, brown, and yellow urine are more commonly encountered, blue-green urine remains a rare and under-researched phenomenon. This case report describes an elderly patient presenting with an incidental finding of blue-green urine, exploring its potential etiologies and emphasizing the importance of clinical history in identifying benign versus pathological causes.

## Introduction

Medical professionals and lay people are often unsure how to interpret abnormal urine colors and whether an abnormality reflects a patient’s underlying health status. This case report reviews an incidental abnormal urine color finding in a patient who presented to the emergency department (ED), considers the likely cause of that patient’s abnormality, and reviews known causes of abnormal urine color.

Under normal physiological conditions, urine varies in color from pale yellow to amber. This color is primarily due to urochrome, a pigment derived from the breakdown of hemoglobin [1]. Deviations from this hue may signal underlying systemic disease, metabolic disorders, renal pathology, or simply benign iatrogenic causes.

Perhaps the most recognizable case of these abnormalities is red-colored urine. For example, hematuria is a clinically significant cause of abnormal urine color and may be microscopically present in 1% to 18% of the population. Potential etiologies for hematuria can range from benign conditions, such as exercise-induced hematuria, to more significant diseases, such as renal cell carcinoma and glomerulonephritis [2]. Similarly, myoglobinuria and hemoglobinuria, both of which result in dark red to brown urine, can indicate physiologic abnormalities such as rhabdomyolysis or intravascular hemolysis, respectively [3]. Pharmacological causes of red urine include warfarin, hydroxocobalamin, and metronidazole. Additionally, certain diets such as ones high in carrots, blackberries, rhubarb, or beets can cause red or pink urine [1]. Lastly, congenital diseases such as porphyria, which can affect up to one in 20,000 individuals, can lead to port-wine-colored urine [4,5].

In contrast, brown, yellow, and orange urine colors have a narrower differential diagnosis. Dehydration can be a common cause of yellow or orange urine color [6]. Bilirubin can also skew the urine toward a brown-orange hue; therefore, diseases that lead to its accumulation can affect urine color [7]. Malignancies of melanocytes, such as melanomas, can cause melanin leakage into the urine, also producing a brown color [6]. Additionally, orange urine can be caused by medications such as phenazopyridine, isoniazid, riboflavin, and sulfasalazine, and brown urine has been associated with medications such as acetaminophen and nitrofurantoin [1].

White or cloudy urine has also been noted in some patients. High amounts of calcium or phosphate can cause these associated changes in color [8]. The most common cause of white urine is chyluria or the presence of chyle in the urine, which can result from a lymphatic fistula or infection with parasitic helminths such as filariasis [9,10]. Infections such as severe UTIs may cause purulent white fluid to enter the bladder, and disseminated infection with tuberculosis (TB) can lead to white urine through the leakage of caseous debris [11].

Cases of blue or green urine have been known to result from iatrogenic, endogenous, and infectious etiologies. Alkaptonuria is an autosomal recessive disease that affects the breakdown of certain amino acids and can lead to dark blue urine [12]. Similarly, Hartnup disease, a disorder that affects intestinal amino acid absorption, can shift urine to a blue hue [13]. Infectious agents such as *Pseudomonas aeruginosa* can produce blue-green urine through the production of specific virulence factors [14,15]. Lastly, pharmacological causes of blue-green urine include medications such as methylene blue, cimetidine, indomethacin, amitriptyline, zaleplon, and propofol [1].

Clinicians presented with these cases of abnormal urine color should obtain a thorough history and physical examination. However, there are also tools that have been made available to providers through the years, which can help narrow the diagnosis. In the mid-20th century, dipstick urinalysis was introduced, a semi-quantitative method that can identify cases of hematuria and bilirubinuria [16,17]. For suspected metabolic disorders, spectrophotometry, also introduced in the mid-20th century, can detect cases of porphyrins or homogentisic acid in patients affected with porphyria or alkaptonuria, respectively [18,19]. More recent technologies, such as high-performance liquid chromatography, can analyze urinary amino acids, aiding in diagnosis, such as Hartnup disease [20]. Similarly, pyocyanin detection via fluorescence spectroscopy can aid in diagnosing Pseudomonas aeruginosa infections when blue-green urine is observed [21].

Although these more thorough methods of investigation may aid in narrowing differential diagnosis, such avenues can remain time-intensive and costly. As such, practitioners must often rely on a subjective interpretation made by a patient or a provider of the particular quality of urine. Unfortunately, the volume of research on urine color varies across specific hues. In particular, blue-green urine has received disproportionately little investigation, leaving a relatively limited differential for providers.

In this case report, we will examine a particular case of a patient with blue-green urine. The urine was collected after the patient reported to the ED after a ground-level fall. This case remains particularly unique because of the series of circumstances that contributed to this patient’s urine color.

## Case presentation

A 77-year-old female presented to the ED after a ground-level fall. The patient was at home when she fell from a standing height without an associated loss of consciousness. The fall itself was unwitnessed by her husband, but EMS was contacted shortly after the event. In transport, the patient remained in stable condition and was alert and oriented. Ketamine was given in transport for acute pain relief. Upon arrival at the ED, the patient was noted to have an open fracture of her right wrist and a transverse laceration. Initial vitals were within normal limits, with a heart rate of 64 beats per minute, a blood pressure of 114/73, a temperature of 97.4°F, and a respiratory rate of 16 breaths per minute. The patient endorsed pain but no numbness or weakness in the affected extremity.

**Figure 1:**
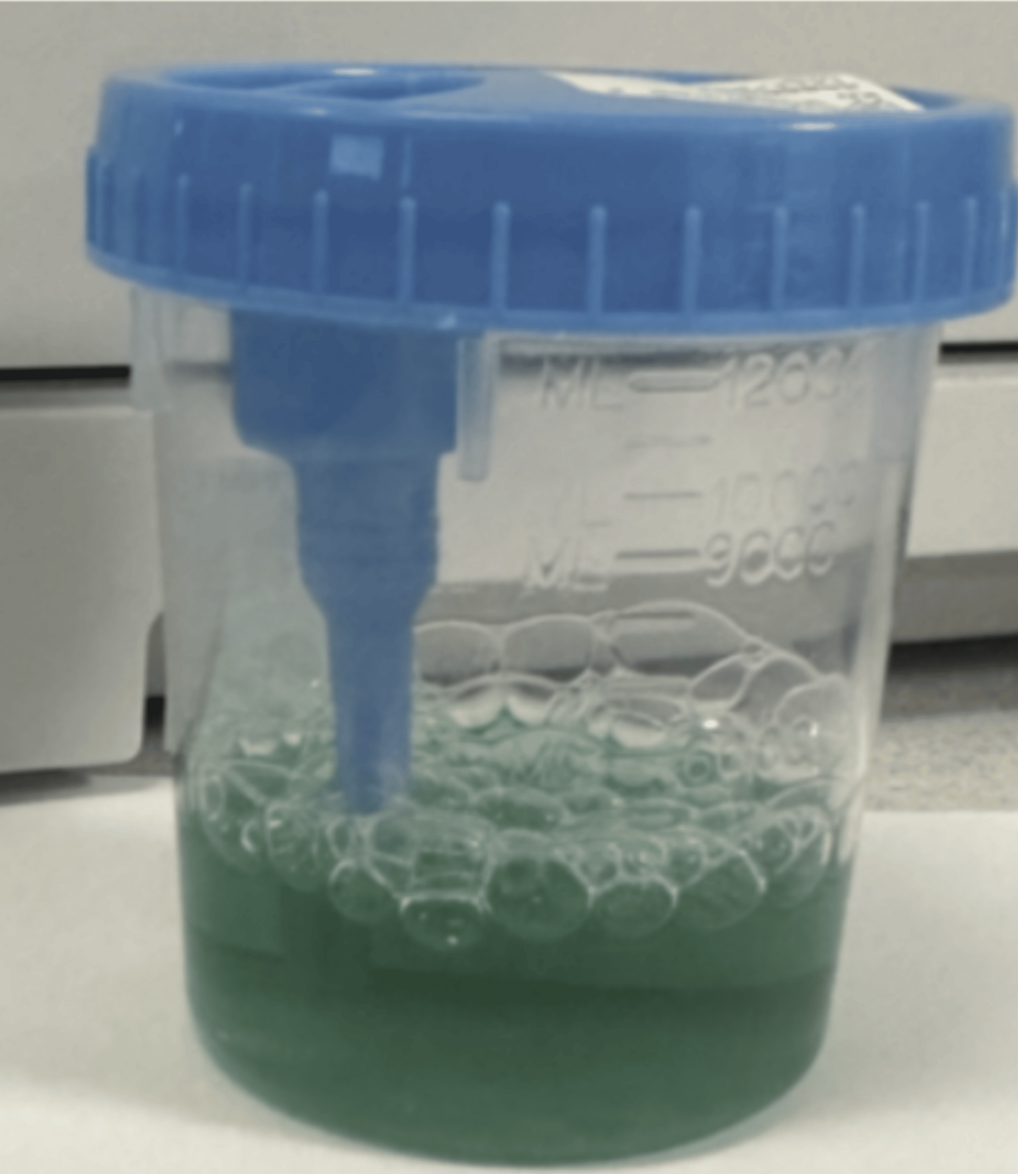
Photo taken by ED staff showing the patient's blue-green urine discoloration.

The patient had a past medical history significant for hypertension and hyperlipidemia, as well as urinary retention, frequent urinary tract infections (UTIs), and prior kidney injury. Notably, past surgical history included a sacral nerve implant for the treatment of urinary retention. Home medications included pregabalin, pantoprazole, ondansetron, methenamine, memantine, magnesium oxide, losartan, duloxetine, buspirone, bupropion, vitamin C, topiramate, and Uro-MP, a medication which contains methenamine, sodium phosphate, phenyl salicylate, methylene blue, and hyoscyamine sulfate. Radiography confirmed an acute fracture dislocation of the right radius. The patient was admitted to the trauma service for open reduction and internal fixation (ORIF) of the fracture. The patient was started on intravenous vancomycin and cefepime consistent with standard treatment for an open fracture. During the ED course, a urine sample was collected and appeared grossly abnormal with a blue-green hue (Figure 1).

A basic laboratory workup was obtained from this patient. Urinalysis results are shown below and were generally unremarkable, showing no signs of acute infection or glomerular dysfunction (Table 1). Additionally, urine electrolytes were mildly low with chloride <20 mmol/L, potassium = 21.7 meq/L, and sodium = 25 mmol/L. Following these tests and successful surgery, the patient was discharged from care and referred to follow-up in the clinic.

**Table 1:** Urinalysis of the patient performed during ED admission. HPF: high power field.

Parameter	Value	Reference range
Urine glucose	Negative	Negative
Urine ketones	Negative	Negative
Urine nitrite	Negative	Negative
Urine protein	Negative	Negative
Urine hemoglobin	Negative	Negative
Urine bilirubin	Negative	Negative
Urine urobilinogen	0.2 mg/dL	<2 mg/dL
Urine color	Blue	Yellow
Urine appearance	Clear	Clear
Urine leukocyte	Negative	Negative
Urine RBCs	0-2/HPF	<2/HPF
Urine WBCs	0-5/HPF	<5/HPF
Squamous epithelial cells	0-5/HPF	<5/HPF
Urine PH	6.5	4.8-7.8
Urine specific gravity	1.010	1.003-1.035

## Discussion

**Figure 2:**
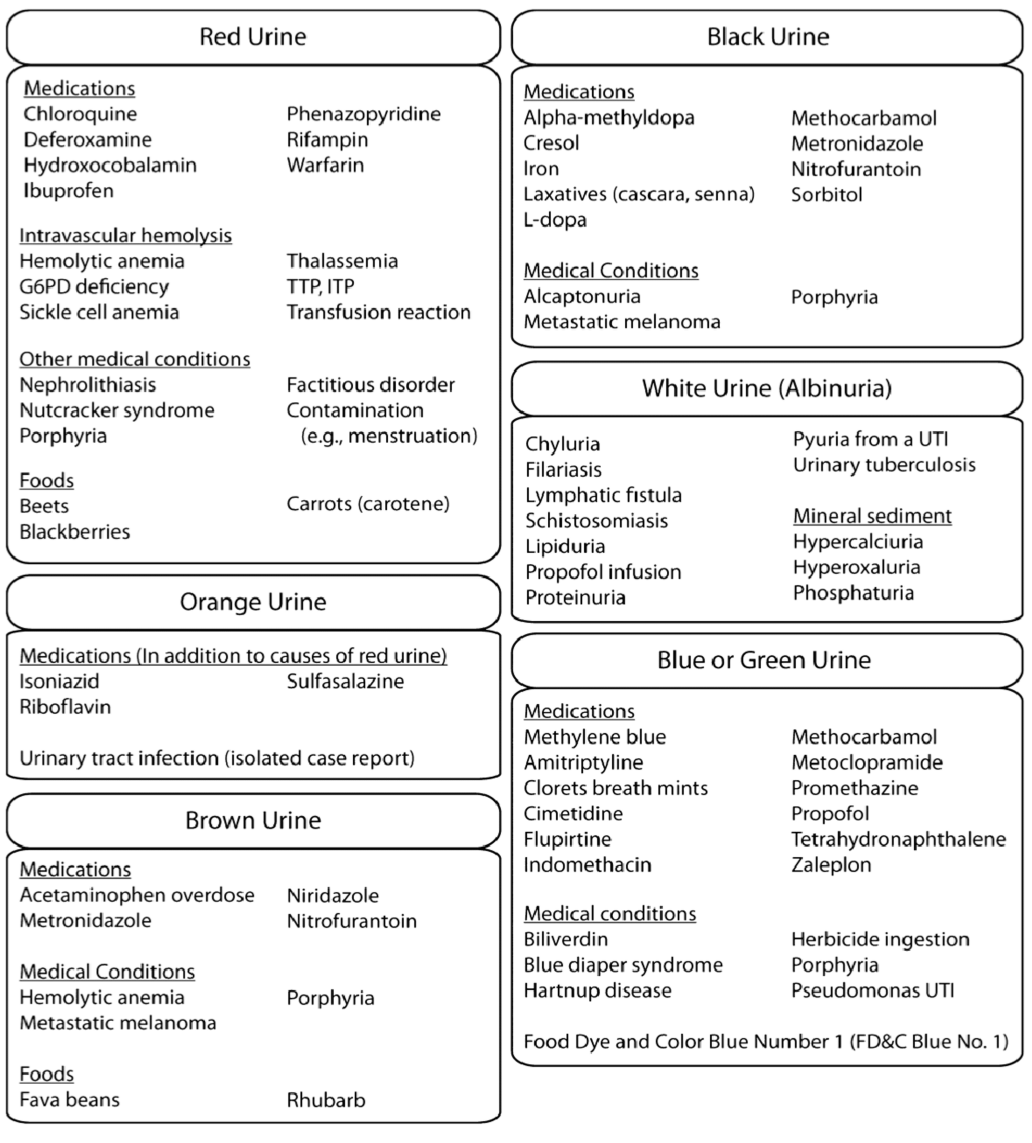
Abnormal urine color chart. G6PD: glucose-6-phosphate dehydrogenase; TTP: thrombotic thrombocytopenic purpura; ITP: immune thrombocytopenic purpura. Source: Figure adapted from Aycock and Kass (2012) [1]. Permission for use of the figure was obtained from Ryan Aycock by Carson Balen.

**Figure 3:**
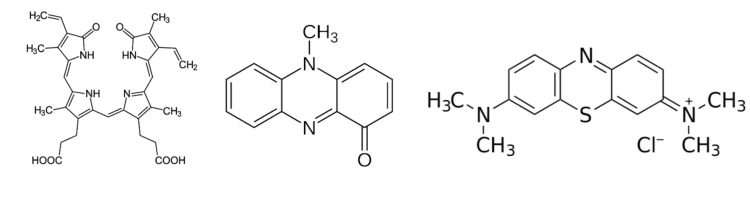
Image of shared conjugated ring structure between biliverdin, pyocyanin, and methylene blue, respectively (left to right). Figure credit: Carson Balen.

There are multiple processes that may have resulted in the patient’s blue-green urine color. A semi-comprehensive summary of different causes of abnormal urine color is shown in Figure 2 [1]. Though rare, diseases that cause a buildup of biliverdin have been known to cause green-hued urine. Biliverdin’s tetrapyrrole structure consists of a conjugated electronic system giving the compound a green color (Figure 3) [22]. Stasis of bile can cause an accumulation of bilirubin precursors, occasionally allowing biliverdin, one of these precursors, to enter systemic circulation. Thus, diseases that cause bile stasis, such as cholestasis, may cause green urine. In the same vein, rare mutations in the processing of biliverdin may lead to its accumulation and resultant green urine [23].

UTI with *P. aeruginosa* leads to production of the virulence factor pyocyanin, which is a pigment that has been known to skew urine color toward a blue-greenish hue. Similarly to biliverdin, pyocyanin is a derivative of a conjugated compound, phenazine, giving it a blue-green color (Figure 3) [14,15]. Numerous medications have also been linked to blue-green urine, such as propofol, methylene blue, cimetidine, amitriptyline, indomethacin, and promethazine [24]. Propofol’s metabolism by cytochrome P450 enzymes to quinol compounds is primarily responsible for its color due, again, to these compounds’ conjugated ring structure [25]. This mechanism is similar to that of promethazine and cimetidine, which also have structural moieties containing conjugated groups, phenols, which may produce a green-hued urine [26].

The 77-year-old patient presented in our case had a history of recurrent UTIs. These prior infections had resulted in prior kidney injury and necessitated the implantation of a sacral nerve stimulator. The patient took a number of medications to help prevent UTIs, including Uro-MP, which contains a compound called methylene blue. Methylene blue has antimicrobial properties due to its ability to function as a redox reagent, which interferes with the bacterial electron transport chain [27]. Methylene blue is also a dye that contains a conjugated aromatic ring responsible for its blue-green color (Figure 3). In normal physiology, methylene blue is reduced by cytochrome P450 enzymes in the liver, yielding leucomethylene blue, a colorless compound [28]. However, when in the urine, the compound is oxidized back to methylene blue, sometimes producing a blue-green urine color [29].

## Conclusions

Abnormal urine color often occurs iatrogenically through medication use. Other causes of abnormal urine color include acquired and inherited pathophysiological disease states. The patient presented in our case was, incidentally, found to have a blue-green colored urine, which likely resulted from her prophylactic UTI prevention medication, Uro-MP, which contained methylene blue. The patient did not have any evidence of a UTI on her urine analysis, thus, an infectious cause of blue-green color change (*Pseudomonas aeruginosa*) was ruled out. In addition, the patient had no prior history of genetic disorders affecting biliverdin or tryptophan and had no known history of right upper quadrant pain, suggesting cholestasis. While rare, abnormal urine colors can appear in clinical encounters, often igniting curiosity and even concern from medical staff and patients. It is important to remember common causes of abnormal urine colors that can often be elucidated via history and physical exam in lieu of more intensive diagnostic tests.
